# Flexible Wolf Pack Algorithm for Dynamic Multidimensional Knapsack Problems

**DOI:** 10.34133/2020/1762107

**Published:** 2020-02-18

**Authors:** Husheng Wu, Renbin Xiao

**Affiliations:** ^1^School of Equipment Management and Support, Armed Police Force Engineering University, Xi'an 710086, China; ^2^School of Artificial Intelligence and Automation, Huazhong University of Science and Technology, Wuhan 430074, China

## Abstract

Optimization problems especially in a dynamic environment is a hot research area that has attracted notable attention in the past decades. It is clear from the dynamic optimization literatures that most of the efforts have been devoted to continuous dynamic optimization problems although the majority of the real-life problems are combinatorial. Moreover, many algorithms shown to be successful in stationary combinatorial optimization problems commonly have mediocre performance in a dynamic environment. In this study, based on binary wolf pack algorithm (BWPA), combining with flexible population updating strategy, a flexible binary wolf pack algorithm (FWPA) is proposed. Then, FWPA is used to solve a set of static multidimensional knapsack benchmarks and several dynamic multidimensional knapsack problems, which have numerous practical applications. To the best of our knowledge, this paper constitutes the first study on the performance of WPA on a dynamic combinatorial problem. By comparing two state-of-the-art algorithms with the basic BWPA, the simulation experimental results demonstrate that FWPA can be considered as a feasibility and competitive algorithm for dynamic optimization problems.

## 1. Introduction

Most research in evolutionary computation focuses on static problems where the entire problem-related data remains stationary through optimization procedure [1–3]. However, numerous real-world optimization problems arising from the uncertainty of future events indeed have a dynamic nature. Changes in dynamic optimization problems (DOPs) may occur in the decision variables, constraints, and objective function [4, 5]. This requires optimization algorithms to not only detect and respond to the change of optima as quickly as possible but also keep track of the changing optima dynamically. Hence, the capability of continuously adapting the solution to a changing environment is necessary for optimization approaches [3, 6]. Therefore, DOPs are more challenging to address than stationary optimization problems.

DOPs can be generally divided into two major fields as combinatorial and continuous [7–9]. Typical combinatorial DOPs include dynamic travelling salesman problem (DTSP) [10], dynamic vehicle routing problem (DVRP) [11], dynamic job-shop scheduling problem (DJSSP) [12], and dynamic knapsack problem (DKP) [13–15]. In fact, many practical problems can be abstracted as a specific type of dynamic multidimensional knapsack problem (DMPK) when multiple dynamic constraints are needed to be tackled, such as task allocation, investment decision, cargo loading, and budget management [9, 16]. Given their wild application and complexity, DMKPs have important theoretical and practical value. Evolutionary algorithms (EAs) and swarm intelligence-based algorithms are expected to perform well on solving both combinatorial and continuous DOPs since evolutionary dynamics in nature also take place in a highly uncertain environment [8, 17, 18].

Wolf pack algorithm (WPA) [19] is a relatively new and promising member of swarm intelligence-based algorithms that model the cooperative hunting behavior of wolf pack. It has been proved an efficient optimizer for solving many nonlinear and complex optimization problems by successful applications in image processing [20], power system control [21], robot path planning [22], and static MKPs [23]. Many derivative versions of WPA also have been designed for solving different problems, such as binary WPA (BWPA) for 0-1 ordinary knapsack problem [24], improved binary WPA (IBWPA) for MKPs [23], and discrete WPA (DWPA) for TSP [25]. In [26], an integer coding wolf pack algorithm (ICWPA) is proposed to cope with the combat task allocation problems of aerial swarm. In [27], the improved WPA (IWPA) is proposed to solve VRP. Despite its high efficiency of binary WPA (BWPA) in solving static MKPs, WPA has not been introduced into the area of DMKPs.

The key issue of handling DOPs using EAs is how to avoid population diversity loss problem and maintain population diversity while tracking the changing global optima [5, 8, 9, 28]. In this regard, a flexible population updating strategy which is capable of introducing and maintaining diversity during execution is designed for BWPA to address the DMKPs in this study. Moreover, the flexible population updating strategy that generates new individuals by making use of the memory of previously found good solutions can be viewed as an explicit memory scheme [29, 30].

Compared with static extensions, there are relatively far less reported publications about DMKPs. It is necessary to develop new solution approaches for addressing DMKPs more efficiently as DMKPs have numerous practical implications. This is one of the main motivations of this study. Secondly, to the best of our knowledge, this is the first study that investigates the performance of BWPA and its improved version (as proposed in this paper) on MKPs in dynamic environments.

The reminder of this paper is arranged as follows: Section 2 provides the literature review and related concepts of DMKPs. The original BWPA and its variant FWPA are discussed in detail in Section 3. While Section 4 conducts the simulation experiment and analyzes the results. Finally, conclusions and some future research issue are given in Section 5.

## 2. Problem Definition and Related Work

In this section, we outline the necessary concepts of DMKPs and overview the related work about the MKPs in dynamic environments.

### 2.1. Definition of the Dynamic Multidimensional Knapsack Problem

MKP is a NP-hard problem and has been wildly used as a combinatorial benchmark problem of EAs and swarm intelligence-based algorithms [31, 32]. The MKP depends on the values of the profits *p*_*j*_, resource consumptions *w*_*kj*_, and the resource constraints *c*_*k*_. As the generalization of the ordinary knapsack problem, MKP is more representative of real-world scenarios because multiple constrains are concerned [33]. The static MKP can be generally formulated as follows [34]:

(1) $$\begin{array}{c} \max f=\overset{n}{\underset{j=1}{\sum }}p_{j}x_{j} \end{array}$$

(2) $$\begin{array}{c} \text{s}.\text{t}.\left\{\begin{array}{c} \overset{n}{\underset{j=1}{\sum }}w_{kj}x_{j}\leq c_{k},\;k\in M=\left\{0,1,\cdots ,m\right\}\\ x_{j}=\left\{0,1\right\},\;j\in N=\left\{0,1,\cdots ,n\right\}, \end{array}\right. \end{array}$$

where *n* is the number of items and *m* is the number of knapsack constrains with capacities *c*_*k*_ for *k* = 1, 2, ⋯, *m*. Each item *j* ∈ **N** requires *w*_*kj*_ units of resource consumption in the *k*^th^ knapsack and yields *p*_*j*_ units of profit upon inclusion. The goal of MKP is to find a subset of all items that yield maximum profit without exceeding the multidimensional resource capacities [34]. All entries are naturally nonnegative. More precisely, without loss of generality, it can be assumed that the following constraints, as defined by (3), are satisfied. If this is not the case, one or more variables could be fixed to 0 or 1.

(3) $$\begin{array}{c} \max\left\{w_{kj}:j\in N\right\}\leq c_{k}<\overset{n}{\underset{j}{\sum }}w_{kj},\;\forall k\in M. \end{array}$$

Dynamic instances of knapsack problems have been proposed before. However, these studies are mainly focused on either only one dimension problem or a cyclic change of the resource constraint [35, 36]. Inspiration from [13, 37], we construct the dynamic MKP by updating all parameters of *w*_*kj*_, *p*_*j*_, and *c*_*k*_ after a predefined simulation time unit using a normally distributed random distribution with zero mean and standard deviation *θ*:

(4) $$\begin{array}{c} p_{j}^{+}=p_{j}\left(1+N\left(0,\theta_{p}\right)\right),\\ w_{kj}^{+}=w_{kj}\left(1+N\left(0,\theta_{w}\right)\right),\\ c_{k}^{+}=c_{k}\left(1+N\left(0,\theta_{c}\right)\right). \end{array}$$

In formula (4), *p*_*j*_^+^, *w*_*kj*_^+^, and *c*_*k*_^+^ denote the updated parameters of MKP when a change occurs after a predefined simulation time units, respectively. The less number of simulation time units yield to more frequent changes and vice versa [13, 37–39]. The number of iterations allocated for each environment is usually adopted as the frequency of changes.

### 2.2. Related Work on DMKPs

In recent years, DMKPs have attracted growing interest from the optimization community with its wide applications and challenging solutions. The related research on DMKPs can be generally summarized as follows:

1. Various dynamic benchmark generators for DMKPs: many generators have been proposed to generate changing environments for MKPs and then translate a well-known static MKP into a dynamic version using specialized procedures. Branke et al. [37] designed a dynamic version of MKP by using a normal distribution to update each parameter of a MKP when a change occurs, as shown in formula (4). Yang and Yao [39] formalized a well-known dynamic problem generator to create required dynamics for a given static combinatorial problem using the bitwise exclusive-or (XOR) operator. This generator is also available for MKPs. Based on a XOR DOP generator, Li and Yang [40] proposed a generalized dynamic benchmark generator (GDBG) that can be instantiated into the binary space, real space, and combinatory space. In addition, the GDBG can present a set of different properties to test algorithms by tuning some control parameters. Rohlfshagen and Yao [38] proposed a new benchmark problem for dynamic combinatorial optimization by taking both the underlying dynamics of the problem and the distances between successive global optima into consideration; the parameters of MKP can be changed over time by some set of difference equations
2. Effects of solution representation techniques for DMKPs: the effects of different solution representations (i.e., weight coding, binary representation, and permutation representation) were compared with a set of DMKPs in [37]. Simulation results revealed that the solution representation affects the algorithms' performance greatly when solving DMKPs and the binary representation performs relatively poor
3. Extensions of DMKPs: there are various versions of DMKPs in terms of the changed parameters of MKPs. In [41], a stochastic 0/1 KP where the value of the items *p*_*j*_ are deterministic but the unit resource consumptions *w*_*kj*_^+^ are randomly distributed was studied. He et al. [42] proposed a more generalized time-varying KP (TVKP) called randomized TVKP (RTVKP) where all parameters of MKPs *p*_*j*_^+^ , *w*_*kj*_^+^, and *c*_*k*_^+^ change dynamically in a random way. Moreover, the dynamic version of MKPs that change its parameters *p*_*j*_^+^ , *w*_*kj*_^+^, and *c*_*k*_^+^ using normal distribution is used as the dynamic benchmark problem of MKPs most wildly [13, 14, 37, 38]
4. Different solution approaches for DMKPs: both EAs and swarm intelligence-based algorithms have been applied to solve DMKPs by adding some strategies to improve their adaptability to dynamic environments. In [42], the elitists model-based genetic algorithm (EGA) was integrated with greedy optimization algorithm (GOA) to handle RTVKPs; the GOA is capable of avoiding infeasible solutions and improving the convergence rate. Ünal [43] adopted the random immigrant-based GA and memory-based GA to solve the DMKPs, respectively. Compared with the random immigrant-based GA, the memory-based GA was proved to be more effective to adapt to the changing environments for DMKPs. Afterward, Ünal and Kayakutlu [14] tested different partial random restarting approaches of parthenogenetic algorithm (PGA) [44] by solving a set of MKPs in dynamic environments. When solving the DMKPs using ant colony algorithm (ACO), Randall [45] updated the pheromone trails indirectly according to the changes made to the solutions during the solution repair period; therefore, partial knowledge of the previous environment is preserved and the adaptability to dynamic environments is enhanced. Baykasoğlu and Ozsoydan [13] proposed an improved firefly algorithm (FA) that introduces population diversity by partial random restarts and the adaptive move procedure. The simulation results showed that the improved FA was a very powerful algorithm for solving both static and dynamic MKPs

## 3. Overview of Binary Wolf Pack Algorithm

Wolf pack algorithm (WPA) is a relatively new swarm intelligence-based optimizer which simulate the collaborative hunting behavior of wolf pack [19]. The basic WPA was originally designed for continuous optimization problems. Due to its simple implementation, robustness, and competitive global convergence performance for high-dimension multimodal functions [19–21], WPA has attracted increasing attention and its various derivative versions for solving discrete problems have been developed in recent years. In [24], Wu et al. proposed a binary WPA (BWPA) based on binary coding of solution to solve the classic 0-1 KPs. Afterward, they modified the BWPA by adding a trying-loading solution repair operator to handle MKPs [23].

Inspired by social hierarchy of biological wolves, individuals in WPA are divided as artificial lead wolf, scout wolves, and ferocious wolves according to their roles during searching optimum. The optimization process of WPA can be generally summarized as scouting, calling, and besieging behavior. In each iteration, the lead wolf can be replaced by other wolves that dynamically own better fitness and the whole population is updated in order to increase diversity. The main operation procedures of the BWPA are summarized as follows:

**Step 1 .**

Initialize the parameters of algorithm step coefficient *S*, distance determinant coefficient *d*_near_, maximum number of repetitions in scouting behavior *T*_max_, and population renewing proportional coefficient *β*. Randomly initialize the position of artificial wolves in *N* × *n* Euclidean space, where *N* is the number of wolves and *n* is the number of variables, the position of artificial wolf *i* is *X*_*i*_ = {*x*_*i*1_, *x*_*i*2,_ ⋯ , *x*_*ij*_, ⋯, *x*_in_}. As for MKP, *X*_*i*_ is a *n* bit binary string and represents a potential solution. *Y*_*i*_ = *f*(*X*_*i*_) denote the objective function value of the wolf *i*. The wolf *X*_lead_ with best objective function value *Y*_lead_ = max{*Y*_*i*_} is selected as the lead wolf of the first generation.

**Step 2 .**

Scouting behavior models the board search of prey in wolf pack's hunting behavior under the command of lead wolf. Except the lead wolf, the rest *n* − 1 wolves act as the scout wolves to take the scouting behavior by implementing the moving operator Θ, respectively, until *Y*_*i*_ > *Y*_lead_ or the scouting repetition number *T* reaches *T*_max_, then go to Step 3.

If *Y*_*i*_ > *Y*_lead_, the scout wolf *i* replaces the role of previous lead wolf and acts as the new lead wolf; Elseif *Y*_*i*_ ≤ *Y*_lead_, the scout wolf *i*, respectively, takes a step towards *h* different directions and move to the best direction *p*∗ (i.e., *Y*_*ip*∗_ = max{*Y*_*ip*_}). *h* is a positive integer that is randomly selected in the interval of [*h*_min_, *h*_max_]. After taking a step towards the *p*^th^ scouting direction (*p* ∈ **H**, **H** = {1, 2, ⋯, *h*}), the position of the scout wolf *i* is updated by

(5) $$\begin{array}{c} X_{i}^{p}=\Theta \left(X_{i},M_{a},{\text{step}}_{a}\right), \end{array}$$

where *X*_*i*_ and step_*a*_ denote the position and step size of the scout wolf *i*, respectively. *M*_*a*_ = {1, 2, ⋯, *m*}. The function of moving operator Θ(**X**_*i*_, **M**_**a**_, step_*a*_) is updating the *X*_*i*_ by reversing the step_*a*_ bits values which are randomly selected from *M*_*a*_.

**Figure 1:**
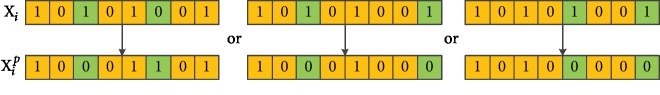
An illustration of moving operator.

Assuming that *X*_*i*_ = {1, 0, 1, 0, 1, 0, 0, 1}, *M*_*a*_ = {3, 6, 8}, and step_*a*_ = 2, the reserving from *X*_*i*_ to **X**_*i*_^*p*^ by moving operator Θ(**X**_*i*_, **M**_**a**_, step_*a*_) can be illustrated as Figure 1.

**Step 3 .**

Except the lead wolf, the rest *n* − 1 wolves secondly act as the ferocious wolves in calling behavior. In order to hunt the prey, the lead wolf commands the ferocious wolves to gather towards its position *X*_lead_ by howling. The position of the ferocious wolf *i* is updated by

(6) $$\begin{array}{c} X_{i}^{\text{new}}=\Theta \left(X_{i},M_{b},{\text{step}}_{b}\right), \end{array}$$

where **X**_*i*_^new^ and step_*b*_ denote the updated position and step size of the ferocious wolf *i*, respectively. **M**_*b*_ is the set of bits with different values between *X*_lead_ and *X*_*i*_. Θ is the same moving operator as defined in Step 2.

If *Y*_*i*new_ ≥ *Y*_lead_, the ferocious wolf *i* replaces the previous lead wolf and restarts the calling behavior; otherwise, the ferocious wolf *i* continues running until *d*_is_ ≤ *d*_near_, then go to Step 4, where *d*_is_ indicates the distance between *X*_lead_ and *X*_*i*_.

**Step 4 .**

After calling behavior, the wolves approach and surround the prey, then the whole wolf pack attack and capture the prey successfully. The position of the wolf *i* is updated by

(7) $$\begin{array}{c} X_{i}^{\text{new}}=\Theta \left(X_{i},M_{c},{\text{step}}_{c}\right), \end{array}$$

where **X**_*i*_^new^ and step_*c*_ denote the updated position and besieging step size of the wolf *i*, respectively. **M**_*c*_ and Θ are the same as that defined in Step 3. The relationships between step_*a*_, step_*b*_, and step_*c*_ are described as follows:

(8) $$\begin{array}{c} \left\{\begin{array}{c} {\text{step}}_{a}=\mathrm{rand}\mathrm{int}\left[{\text{step}}_{c},S\right],\\ {\text{step}}_{b}=\mathrm{rand}\mathrm{int}\left[{\text{step}}_{c},2S\right], \end{array}\right. \end{array}$$

where step_*c*_ is commonly set to 1; randint indicates a randomly selected integer in this interval.

**Step 5 .**

Update the position of wolf pack with population renewing proportional coefficient *β*.

**Step 6 .**

Output the position and function value of lead wolf (i.e., the optimal solution) when termination condition is satisfied, otherwise go to Step 2. The pseudocode of BWPA is illustrated in Algorithm 1.

## 4. Proposed Flexible Wolf Pack Algorithm

Flexibility is the ability to respond to changing environments effectively. Flexible wolf pack algorithm (FWPA) does not pursue the ultimate convergence of the population, but to maintain the diversity of the population throughout the evolution process, that is, to maintain a strong ability to open up new solution space, which of course should be combined with elite retention strategies. In this section, a flexible population updating strategy based on convergence situation is designed for FWPA to develop its capability of adapting to changing environments.

### 4.1. Original Population Updating Strategy in BWPA

For the BWPA, there are two cases of updating the population: population updating in normal situation and catastrophic situation.

Population updating in normal situation indicates that *R*(*R* = randint[*N*/(2*β*), *N*/*β*]) artificial wolves with worst objective function values are deleted, while *R* new wolves are generated near the lead wolf by

(9) $$\begin{array}{c} X_{\text{new}}=\Theta \left(X_{\text{lead}},M,\left\lfloor L_{1}\right\rfloor \right),\;L_{1}=\frac{1}{4}\cdot \frac{\exp\left(z\left(g\right)\right)-\exp\left(-z\left(g\right)\right)}{0.1\exp\left(z\left(g\right)\right)+2\exp\left(-z\left(g\right)\right)}, \end{array}$$

where *X*_lead_ denotes the position of the lead wolf, *M* = {1, 2, ⋯, *n*}, and *z*(*g*) = 10*g*/MaxGen − 5; ⌊·⌋ represents that rounding down of *L*_1_ to an integer.

Population updating in catastrophic situation indicates that *R* artificial wolves are randomly selected and deleted from the whole population when the best objective function value is not updated in *t*_max_ continuous iterations, then *R* new wolves are reproduced by

(10) $$\begin{array}{c} X_{\text{new}}^{*}=\Theta \left(X_{i},M,\left\lceil L_{2}\right\rceil \right),\;L_{2}=\left\{\begin{array}{c} \frac{k_{1}\left(f\left(X_{\text{lead}}\right)-f\left(X_{i}\right)\right)}{f\left(X_{\text{lead}}\right)-f_{\text{avg}}},f\left(X_{i}\right)\geq f_{\text{avg}},\\ k_{2},f\left(X_{i}\right)<f_{\text{avg}}, \end{array}\right. \end{array}$$

where ⌈·⌉ represents that rounding up of *L*_2_ to an integer, *f*_avg_ is the average fitness value of whole population. *k*_1_ and *k*_2_ are commonly set to 2 and 4, respectively.

### 4.2. Flexible Population Updating Strategy

The original population updating strategy helps to increase the population diversity to some degree; however, it may also lead to two problems: (1) *L*_1_, *L*_2_, and the average fitness value of whole population *f*_avg_ are evaluated in each generation so that the computational cost increases. (2) In the catastrophic situation, the best objective function value is not updated after *t*_max_ continuous generations, which can be judged that the lead wolf has fallen into a local optimum. Generating *R* new wolves based on the previous randomly selected wolves has a tiny effect on jumping out the current local optima, because the updated wolves may gather to the previous lead wolf with a large probability. Therefore, the original population updating strategy is ineffective to introduce or maintain population diversity in a catastrophic situation.

Based on the above analysis, we design a simpler and efficient population updating strategy using the Cauchy distribution random number. Cauchy distribution is a well-known continuous probability distribution. Its probability density function and distribution function are presented by formulas (11) and (12), respectively.

(11) $$\begin{array}{c} f\left(x,z,\tau \right)=\frac{1}{\pi \tau }\cdot \frac{1}{1+{\left(\left(x-z\right)/\tau \right)}^{2}},-\infty <x<+\infty ,\tau >0, \end{array}$$

(12) $$\begin{array}{c} F\left(x,z,\tau \right)=\frac{1}{2}+\frac{1}{\pi }\arctan\left(\frac{x-z}{\tau }\right), \end{array}$$

where *z* is the positional parameter and *τ* is the scaling or shape parameter. Cauchy distribution is named the standard Cauchy distribution *C* (0,1) when *τ* = 1 and *z* = 1.

The Cauchy random numbers can be obtained by converting formula (12) to its inverse function (13), where *F*(*x*) ∈ *u*(0, 1).

(13) $$\begin{array}{c} x=z+\tau \tan\left(\pi \left(F\left(x\right)-0.5\right)\right). \end{array}$$

**Figure 2:**
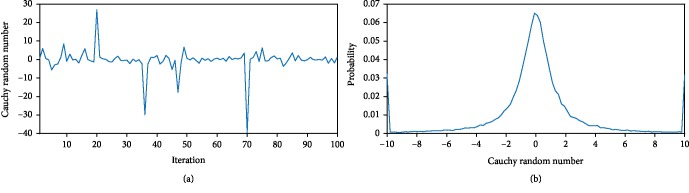
(a) Distribution of Cauchy random number with iterations. (b) Probability of Cauchy random number.

The distribution of the Cauchy distribution random numbers with iterations is shown as Figure 2.

As can be seen from Figures 2(a) and 2(b), the Cauchy random numbers consist of a few mutation numbers and many smoothly fluctuating numbers. Such distribution property is available for generating a few mutant wolves when updating their position in the search process. Therefore, the flexible population updating strategy can be formulated as

(14) $$\begin{array}{c} X_{\text{new}}=\left\{\begin{array}{c} \Theta \left(X_{\text{lead}},M,C_{1}\right),t>t_{\max},\\ \Theta \left(X_{\text{lead}},M,C_{2}\right),t\leq t_{\max}, \end{array}\right. \end{array}$$

where *C*_1_ = ⌈|*x*|⌉ and *C*_1_ = *C*_2_/*μ*, *X*_new_ denotes the position of new generated wolves, and *μ* is the correlation coefficient that binds the population updating in both normal and catastrophic situation together.

The flexible population updating strategy can be described as follows: in normal situation *t* ≤ *t*_max_, similar to original population updating strategy, *R* worst wolves are deleted and then *R* new wolves are generated based on the position of lead wolf. In catastrophic situation *t* > *t*_max_, contrary to normal situation, *R* current best wolves are deleted. The pseudocode of flexible population updating strategy is shown as Algorithm 2.

In fact, *C*_1_ and *C*_2_ can be viewed as the distance between the reinitialized wolf and the previous lead wolf. The larger *C*_1_ and *C*_2_ yield to new wolves that are more different from the previous lead wolf. Therefore, *C*_1_ is larger than *C*_2_ when *μ* subjects to (0,1). The new generated wolves are close to the previous lead wolf in normal situation, and the convergence rate can be accelerated because the positive individual informant is reused, while the new generated wolves are relatively far away from the previous lead wolf in catastrophic situation, so that the negative informant is deleted and the population diversity is increased consequently. The idea of this dynamic population updating strategy compromises the merits of *Partial restart* [46–48] and *Memory scheme* [49, 50]. The dynamic population updating strategy is inferior to the original ones in terms of increasing population diversity and previous informant reusing. The capability of adapting the dynamic environments of BWPA is developed.

### 4.3. Adapting in Changing Environments

All swarm intelligence-based algorithms are initially designed for converge to the optima quickly and precisely. However, when solving DOPs, the capability of adapting to changing environments (i.e., detecting and tracking the changing optima quickly) is necessary. The efficient approach of increasing/maintaining population diversity is significant for enhancing the adaptation capability. However, too high level of diversity will not always lead to better performance for an algorithm. The *knowledge transfer* and *diversity maintenance* should be well balanced.

In this study, the proposed flexible population updating strategy is capable of generating new wolves at each generation, so the diversity loss problem can be well addressed. After generating the new wolves, the fitness values of the whole population are reevaluated at each generation; the changed optima can be detected and tracked when all parameters of an MKP change. Moreover, the population is updated with the use of previous positive information, which is also beneficial for converging to the new optima quickly. Therefore, any other dynamic change detecting method is required.

### 4.4. Design of the FWPA for DMKP

The pseudocode of proposed FWPA is shown as Algorithm 3. To some extent, all dynamic methods try to make balance between diversification (global search), intensification (local search), and the balance between accuracy and speed. FWPA shows a good performance on both of them.

## 5. Simulation Experiments

To verify the performance of FWPA, we conduct both the static and dynamic experiments using a set of MKP benchmarks.

### 5.1. Experimental Data Set

As for stationary environment, we select 9 different benchmark problems with different difficulty levels available on the OR-LIBRARY website (http://people.brunel.ac.uk/mastjjb/jeb/orlib/files). The items of these instances, *n*, range from 100 to 500, and the constraints, *m*, vary from 5 to 30. These problems were also previously used in [14, 51–53]. We express these instances by the notation *m*.*n*.*i* which indicates the *i*th instance with *m* constraints, *n* items. For example, 10.250.00 is the first instance of *mknapcb5.txt* with 10 constraints, 250 items, and tightness ratio of 0.25.

As for the dynamic environment, similar to [13, 37], dynamic instances of MKP are designed by updating the parameters after a predefined simulation time units as defined in Section 2.1. The instance of 10.250.00 was adopted here as the initial and basic environment to generate the changing environments. After the change occurs, the parameters are updated by formula (4).

### 5.2. Experimental Setup and Parameter Setting

For static experiments, the correlation coefficient *μ* is set to 0.5, 0.75, 1, and 2, respectively, to measure its effect on the performance of the improved BWPA. Two state-of-the-art algorithms that have been used to solve the MKPs are used for comparisons; they are chaotic binary particle swarm optimization with time-varying acceleration coefficients (CBPSOTVAC) [51] and parthenogenetic algorithm (PGA) [14]. The parameters of the algorithms were set as shown in Table 1. For each algorithm and each problem, 30 independent runs with 1000 iterations are implemented; the population sizes of all algorithms are equal to 100. The best solution found (Best), the mean of the solutions (Avg.), and the standard deviation of all solutions (Std) overruns are used as the performance measures.

For dynamic experiments, the standard deviation *σ* of normal distributions of each parameter is assumed to be equal. *σ* reflects the severity of dynamic changes, so two different values of *σ*_*p*_ = *σ*_*w*_ = *σ*_*c*_ = 0.05 and *σ*_*p*_ = *σ*_*w*_ = *σ*_*c*_ = 0.1 are set, respectively, to test the proposed algorithm's capability of adapting to the different dynamic environments. In this study, for each algorithm and each problem, 30 independent runs with 2000 iterations are implemented, and a series of 200 iterations is adopted as the frequency of changes. Therefore, 10 different environments were generated using the basic environment (i.e., the instance of 10.250.00). The average best-of-generation was used to measure the algorithm's ability of finding a better solution at each generation in dynamic environments.

Both static and dynamic experiments were executed using the MATLAB software with a personal computer bundled with Intel i7 1.6 GHZ processor and 8 GB RAM.

### 5.3. Results on Stationary Environment

Results on stationary environment are shown in Table 2. The best results of each instance achieved by algorithms are denoted in bold.

According to the results presented in Table 2, FWPA proves inferior to the other three approaches in the majority of the problems in terms of Best, Avg., and Std. With the introduction of dynamic population updating strategy, the proposed algorithm enables to maintain the population diversity and enhance the capability of jumping out of the local optima. Therefore, FWPA can find better solutions and the efficiency of the proposed strategy is proved.

From the comparison of the different versions of FWPA that own different values of *μ*, it can be seen that the FWPA with *μ* = 0.75 performs best, and the performance of the algorithms with *μ* = 0.5, 1, and 2 is similar to BWPA. For each test instance, the FWPA with *μ* = 0.75 achieves best results in terms of Best and Avg. Therefore, the parameter *μ* might affect the performance of FWPA crucially. In the following dynamic experiments, the parameter *μ* is set to 0.75.

For Std, the FWPA with *μ* = 0.75 achieves the better results than the compared algorithms in Inst. 10.100.14, 10.250.14, 10.500.0, and 30.100.0, which shows that the proposed algorithm has a good stability.

### 5.4. Results on Dynamic Environment

Results of average best-of-generation on dynamic environments that are generated by the instance 10.250.00 are shown in Table 3. An efficient algorithm is expected to quickly adapt to new environments and track the moving optima. From the results presented in Table 3, it can be seen that the proposed algorithm outperforms the compared algorithms for *σ* = 0.05 and 0.1. By partially restarting new wolves based on the memory of previous stored informant, the proposed algorithm is capable of tracking the changing optima quickly by efficiently maintaining/introducing the population diversity.

By comparing the results when *σ* = 0.05 and 0.1, which reflect the severity of the change between two dynamic environments, it can be seen that the differences of two consecutive environments become larger with the increase of *σ*. The proposed algorithm is capable of tracking the changing optima quickly and find better results than the other algorithms; this situation can attribute to the powerful capability of opening up new solution space using the dynamic population updating strategy.

**Figure 3:**
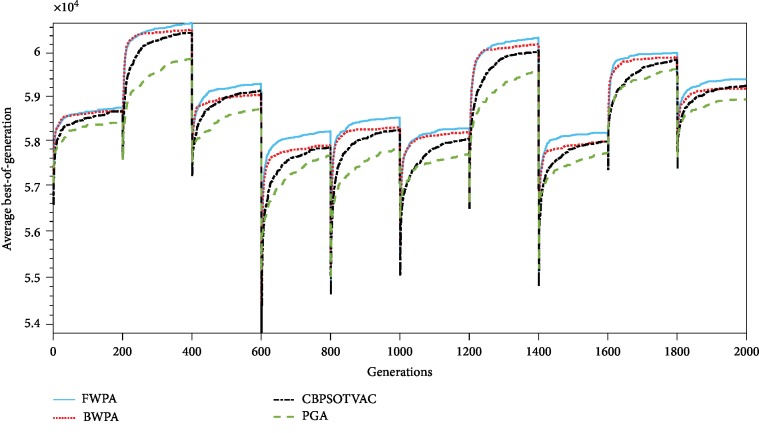
Convergence of algorithms on dynamic environments when *σ* = 0.05.

**Figure 4:**
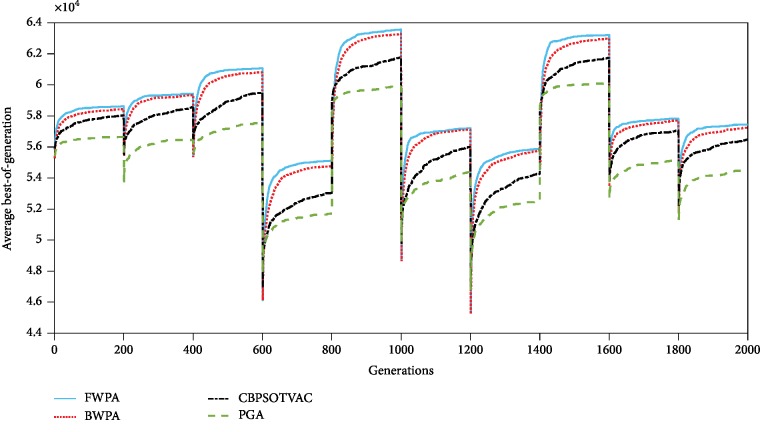
Convergence of algorithms on dynamic environments when *σ* = 0.1.

Convergence graphs of the four algorithms when *σ* = 0.05 and 0.1 are presented in Figures 3 and 4, respectively. For each change, the FWPA can achieve best results. It is apparent from the figures that the proposed algorithm has more efficient capability of adapting the dynamic environments.

### 5.5. Statistical Verification

The statistical results of comparing algorithms by a one-tailed test with 98 degrees of freedom at a 0.05 level of significance are given in Table 4. In Table 4, the *t*-test result regarding FWPA, BWPA, PGA, and CBPSOTVAC is shown as “+,” “~,” and “-” when one algorithm is insignificantly better than, insignificantly worse than, significantly better than, and significantly worse than the other one, respectively.

From the statistical verification presented by Table 4, we can conclude that FWPA outperforms the other three algorithms for both dynamic and stationary environments. This result demonstrates the effectiveness of the dynamic population updating strategy.

## 6. Conclusions and Future Work

This paper presents a flexible BWPA (FWPA) by designing a novel and simpler flexible population updating strategy. The proposed flexible population updating strategy aims at addressing the problem of lack diversity for the WPA during the procedure of solving dynamic optimization problems. In fact, the flexible population updating strategy is a hybridization of *Partial restart* and *Memory scheme* strategy. The simulation experiments on a set of static MKPs prove the effectiveness of the proposed algorithm. Moreover, the simulation experiments on dynamic MKP instances demonstrate that the FWPA is capable of tracking the changing optima quickly and converge to a good solution.

To the best of our knowledge, this paper constitutes the first paper on the combinatorial dynamic optimization problems of WPA. Another contribution of the study is extending the family of approaches for dynamic optimization.

One of the future work is conducting a comparative study with the advanced algorithms such as jDE, SaDE, and HyperMutation GA which were designed particularly for dynamic optimization problems. Moreover, the FWPA will be applied on various combinatorial dynamic optimization problems such as DTSP, DVRP, and DJSSP. Continuous dynamic optimization is also an expected research issue for WPA. Besides, as a relatively new metaheuristic algorithm, WPA has room for developing the performance of solving dynamic optimization problems in the long run. The issues of addressing the dynamic optimization problems more efficient for WPA can be summarized as follows:

1. A more powerful capability of detecting and tracking the dynamic events
2. Faster convergence rate along with the capability of being stuck in local optima
3. Taking advantages of gathered evolutionary information to decrease computational cost and adapting the changing environment more efficiently
4. Self-adaptive parameters tuning performance to decrease the difficulty of implementation

## Figures and Tables

**Algorithm 1:**
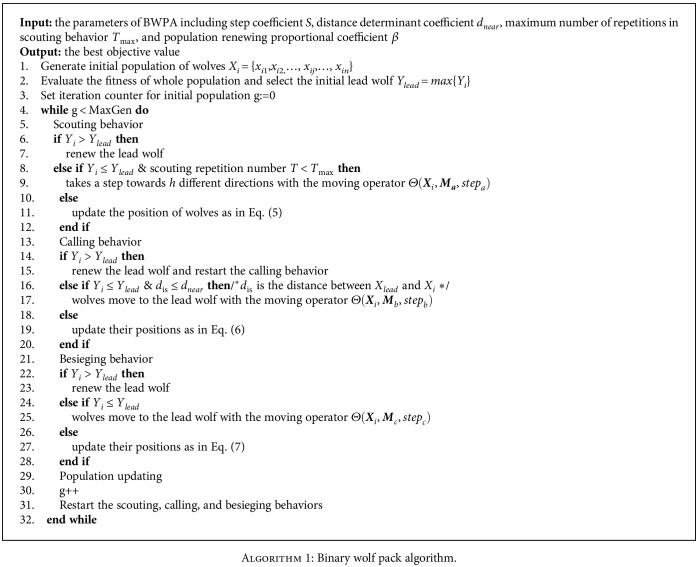
Binary wolf pack algorithm.

**Algorithm 2:**
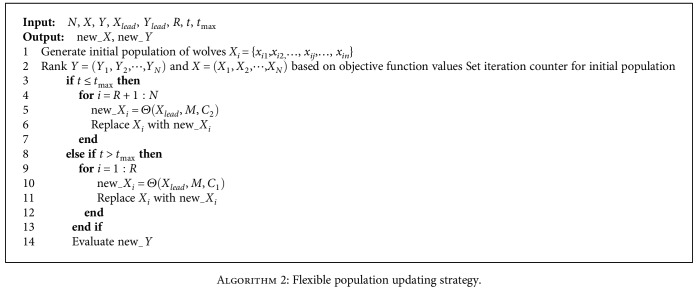
Flexible population updating strategy.

**Algorithm 3:**
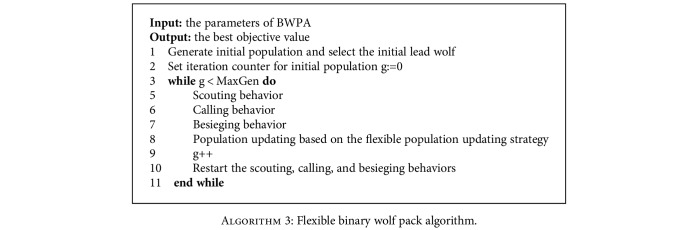
Flexible binary wolf pack algorithm.

**Table 1:** Parameters of the algorithms.

Algorithm	Main parameters
PGA	Elitism rate 0.01, insert rate 0.3, reserve rate 0.3, swap rate 0.3.
CBPSOTVAC	Inertial weight *w*_max_ = 1.5, *w*_min_ = 0.5, acceleration coefficient *c*_1*i*_ = *c*_2*f*_ = 2.5, *c*_2*i*_ = *c*_1*f*_ = 0.5, *V*_min_=4.
BWPA	Step coefficient *S* = 2, distance determinant coefficient *d*_near_ = 4, maximum number of repetitions in scouting behavior *T*_max_ = 10, population renewing proportional coefficient *β* = 2.
FWPA	The parameters are the same as those of BWPA, *μ* = 0.5/0.75/1/2.

**Table 2:** Experimental results on stationary environments for MKPs.

Inst. (best known)		PGA	CBPSO TVAC	BWPA	FWPA			
					*μ* = 0.5	*μ* = 0.75	*μ* = 1	*μ* = 2
5.500.0 (120148)	Best	117365	118242	119406	119567	**119748**	119540	119421
	Avg.	116554	118104.6	119297.4	119333.6	**119413.6**	119361	119219.4
	Std	646.30	311.97	**89.90**	180.66	222.94	445.88	184.88

5.500.14 (218966)	Best	217404	217237	218130	218257	**218474**	218444	218325
	Avg.	216717	216815.6	21783.6	21792.5	**218163**	21832.6	218104.6
	Std	567.87	257.11	435.19	352.54	239.09	**107.92**	221.07

10.100.0 (23064)	Best	22947	23055	22961	22925	**23057**	23055	22961
	Avg.	22879.6	**22958.2**	22843.8	22830.8	22850.8	22926.4	22886.2
	Std	55.76	194.35	93.14	**83.60**	124.41	109.04	68.20

10.100.14 (41884)	Best	41572	41646	41727	41748	**41791**	41767	41737
	Avg.	41491	41525	41655.6	41643.4	**41704**	41688	41655.2
	Std	82.76	112.91	74.56	66.98	**50.90**	86.52	79.41

10.250.0 (59187)	Best	57943	58338	58846	58577	**58904**	58736	58714
	Avg.	57582.8	58132.8	58670.4	58333	**58564.8**	58522.6	58472.6
	Std	323.03	**139.42**	151.10	265.13	236.87	133.49	258.26

10.250.14 (108485)	Best	107369	107546	107932	108081	**108142**	108090	107853
	Avg.	107118.4	107067	107698	107914	**107958.2**	107859.2	10770.7
	Std	183.70	486.99	168.55	159.78	**108.08**	223.03	143.45

10.500.0 (117821)	Best	114842	115067	116159	116218	**116840**	116574	116389
	Avg.	114250.8	114852.4	116066.6	115896.2	**116631.4**	116134.6	116126.6
	Std	645.53	357.39	247.15	268.64	**150.82**	334.27	308.00

30.100.14 (41058)	Best	40866	40917	40954	40957	**41058**	40912	40922
	Avg.	40751.8	40747.8	40857.8	40817.6	**40920.4**	40797.6	40843.6
	Std	93.86	144,72	**58.17**	91,75	100.65	74.92	80.97

30.250.0 (56842)	Best	55374	55921	56194	55851	**56266**	56060	56057
	Avg.	54827	55719.4	55916.4	55554	**56069.4**	55818.4	55827.8
	Std	445.88	**209.81**	219.60	294.70	234.40	300.21	223.32

**Table 3:** Experimental results on dynamic environments for MKPs.

	Env.	PGA	CBPSOTVAC	BWPA	FWPA (*μ* = 0.75)
*σ* = 0.05	1	58131.4	58612.1	58667.6	**58736.8**
	2	59507.5	60451.9	60540.2	**60693.6**
	3	58393.3	59075.9	59028.5	**59283.7**
	4	57377.3	57825.1	57885.6	**58205.5**
	5	57536.6	58152.1	58292.3	**58508.9**
	6	57352.9	57978.0	58187.3	**58267.2**
	7	59151.5	60019.2	60208.9	**60357.7**
	8	57425.0	57968.8	57968.4	**58169.5**
	9	59327.7	59781.1	59907.0	**60002.7**
	10	58601.6	59199.0	59195.0	**59399.0**

*σ* = 0.1	1	56192.1	57558.4	58371.0	**58613.6**
	2	56070.7	58026.1	59257.0	**59422.1**
	3	56935.1	58986.9	60776.0	**61069.1**
	4	51729.2	52506.8	54635.9	**55060.1**
	5	58376.2	61149.4	63197.6	**63560.1**
	6	53055.8	55420.5	57063.8	**57213.6**
	7	51145.4	53753.6	55662.7	**55840.1**
	8	58366.9	61091.3	62909.5	**63215.7**
	9	53742.7	56579.1	57636.9	**57824.0**
	10	53224.3	56022.8	57159.0	**57435.7**

**Table 4:** *t*-test results of the algorithms.

*t*-test result	Static									Dynamic	
	1	2	3	4	5	6	7	8	9	*σ* = 0.05	*σ* = 0.1
FWPA-BWPA	+	+	+	+	+	+	+	+	+	+	+
FWPA-CBPSOTVAC	+	+	+	+	+	+	+	+	+	+	+
FWPA-PGA	+	+	+	+	+	+	+	+	+	+	+
BWPA-CBPSOTVAC	+	+	+	+	+	+	+	+	+	~	+
BWPA-PGA	+	+	+	+	+	+	+	+	+	+	+
PGA-CBPSOTVAC	-	-	-	-	-	-	-	-	-	-	-
